# Polyunsaturated fatty acids-rich dietary lipid prevents high fat diet-induced obesity in mice

**DOI:** 10.1038/s41598-023-32851-7

**Published:** 2023-04-05

**Authors:** Yuri Haneishi, Yuma Furuya, Mayu Hasegawa, Hitoshi Takemae, Yuri Tanioka, Tetsuya Mizutani, Mauro Rossi, Junki Miyamoto

**Affiliations:** 1 Department of Applied Biological Science, Graduate School of Agriculture, Tokyo University of Agriculture and Technology, Fuchu-shi, Tokyo 183-8509 Japan; 2 Center for Infectious Diseases Epidemiology and Prevention Research: CEPiR, Tokyo University of Agriculture and Technology, Fuchu-shi, Tokyo 183-8509 Japan; 3 Department of International Food and Agricultural Science, Faculty of International Food and Agricultural Studies, Tokyo University of Agriculture, Setagaya-ku, Tokyo 156-8502 Japan; 4 Institute of Food Sciences, CNR, via Roma 64, 83100 Avellino, Italy

**Keywords:** Biochemistry, Endocrinology, Gastroenterology

## Abstract

Diet is the primary factor affecting host nutrition and metabolism, with excess food intake, especially high-calorie diets, such as high-fat and high-sugar diets, causing an increased risk of obesity and related disorders. Obesity alters the gut microbial composition and reduces microbial diversity and causes changes in specific bacterial taxa. Dietary lipids can alter the gut microbial composition in obese mice. However, the regulation of gut microbiota and host energy homeostasis by different polyunsaturated fatty acids (PUFAs) in dietary lipids remains unknown. Here, we demonstrated that different PUFAs in dietary lipids improved host metabolism in high-fat diet (HFD)-induced obesity in mice. The intake of the different PUFA-enriched dietary lipids improved metabolism in HFD-induced obesity by regulating glucose tolerance and inhibiting colonic inflammation. Moreover, the gut microbial compositions were different among HFD and modified PUFA-enriched HFD-fed mice. Thus, we have identified a new mechanism underlying the function of different PUFAs in dietary lipids in regulating host energy homeostasis in obese conditions. Our findings shed light on the prevention and treatment of metabolic disorders by targeting the gut microbiota.

## Introduction

Diet is the primary factor in host nutrition and metabolism; however, excess food intake causes dysregulation of energy balance and leads to metabolic disorders such as obesity and type II diabetes^1^. Excess food intake, especially in high-calorie diets, such as high-fat (HFD) and high-sugar diets, is considered the greatest risk factor in the development of obesity^2^. Obesity is a complex disease associated with increased inflammatory markers, leading to chronic low-grade systemic inflammation, which is implicated in the development of insulin resistance^3^. Recently, gut microbiota has also been proposed to be involved in the development of metabolic disorders^4^. Lipopolysaccharides (LPS), also known as endotoxins, are membrane components of gram-negative bacteria that induce inflammation by activating toll-like receptor (TLR) 4, which is expressed on immune cells such as macrophages as well as on other cell types including hepatocytes and adipocytes. LPS can also trigger HFD-induced insulin resistance^5^. Additionally, mucosal TLR activation contributes to hepatic steatosis via the TLR adaptor MYD88 expressed in the intestine^6^. Mice with intestinal epithelial cell-specific MYD88 deletion fed an HFD have improved glucose homeostasis and decreased hepatic lipid content compared with wild-type mice^6^. Moreover, obesity is associated with LPS leakage across the intestinal epithelial barrier, resulting in the potent induction of systemic inflammation^7^.

Dietary lipids can alter gut microbial compositions in obese individuals and mouse models. Comparing mice fed a variety of diets (low-fat diet and diet containing high levels of either saturated lipids, ω6 polyunsaturated fatty acids (PUFAs) or ω3 PUFAs) revealed that diets with saturated lipids or ω6 PUFAs induced weight gain, but only saturated lipids caused increased insulin resistance, colonic permeability, and mesenteric fat inflammation^8^. The gut microbiota composition differed from other groups in mice fed a low-fat diet or a ω3 PUFA diet, but was similar in mice fed either a saturated fat diet or a ω6 PUFA diet. Another study showed that ω3 PUFA-enriched flaxseed oil reduced the ratio of Firmicutes/Bacteroidetes in type 2 diabetic rats^9^. In addition, the fish oil administration induced beneficial bacteria, such as *Lactobacillus*, *Akkermansia muciniphila*, and *Bifidobacterium*, and gut bacteria differences contributed to obesity phenotypic alteration in fish-oil-fed mice compared with lard-fed mice^10^. Lard is rich in saturated fatty acids, while fish oil is enriched in the ω3 PUFAs, docosahexaenoic acid (DHA) and eicosapentaenoic acid (EPA). Recently, Qin et al. reported that fish oil extracted from *Coregonus peled* facilitated the growth of *Bifidobacterium* and *Adlercreutzia*, thereby improving recurrent obese phenotypes in mice^11^.

Furthermore, PUFAs as free fatty acids (FFAs), including DHA and EPA, may promote glucagon-like peptide-1 (GLP-1) secretion and attenuate chronic inflammation in peripheral tissues^12,13^. Colon-specific delivery of DHA– and EPA–induced GLP-1 and insulin release with subsequent reductions in glucose concentrations^14^. Moreover, long-term intracolonic DHA administration for 4 weeks increased plasma GLP-1 levels and decreased non-fasting blood glucose concentrations^15^. In humans with abdominal obesity and insulin resistance, ingestion of a Mediterranean diet rich in olive oil for 28 days resulted in significantly higher postprandial GLP-1 blood concentrations^16^. Compared to a diet enriched in saturated fatty acids, consumption of the Mediterranean diet also improved insulin sensitivity, which could be responsible for the decreased insulin secretion as well as fasting and postprandial blood glucose concentrations^15^. Moreover, recent research suggests that DHA can improve insulin signaling in HFD-fed mice by affecting the gut-adipose axis^17^. Recently, concerns have emerged that a ω6 PUFA-containing diet exacerbated the risk of intestinal diseases^18^. These concerns also stem from evidence that traditional dietary habits and lifestyle unique to the Mediterranean diet lower the incidence of chronic diseases and improve longevity^19^. However, the mechanisms underlying the beneficial effects of PUFA-enriched oils, but not FFAs in obesity needed to be investigated.

In this study, we investigated whether a PUFA-enriched diet could prevent HFD-induced obesity by modulating the gut microbiota. We compared phenotypical and biochemical parameters in obese mice fed diets enriched in either soybean oil, linseed oil, fish oil, or olive oil. Moreover, we performed 16S rRNA analysis to explore the role of the gut microbiome in these beneficial functions of PUFAs. Our study will aid the prevention and treatment of metabolic disorders by targeting the gut microbiota.

## Results

### Polyunsaturated fatty acids improve glucose metabolism via GLP-1 secretion

**Figure 1:**
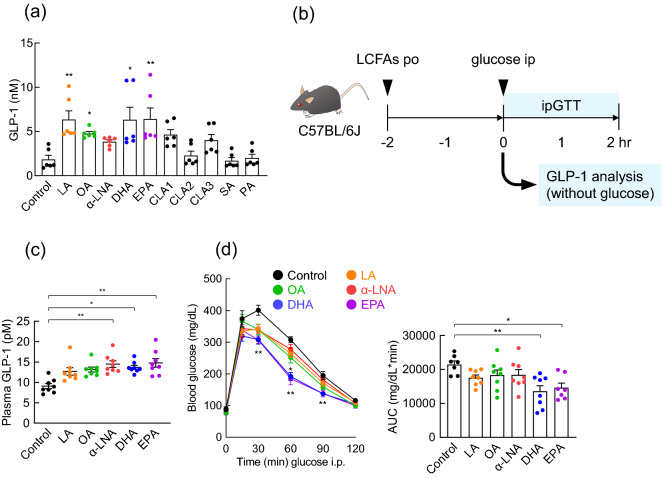
Acute administration of PUFAs improved glucose metabolism by promoting GLP-1 secretion. (**a**) GLP-1 secretion in the STC-1 cells treated with each LCFA (n = 6 per group). **P < 0.01; *P < 0.05, vs. Control (Dunn’s test). (**b**) Experimental scheme. (**c**) Each mouse was administered LCFAs by oral gavage. After 2 h, the plasma GLP-1 levels were measured (n = 8 mice for each group). **P < 0.01; *P < 0.05, vs Control (Dunn’s test). (**d**) Each mouse was administered LCFAs by oral gavage. ipGTT was evaluated (*left panel*) and area under the curve (AUC) of ipGTT was calculated (*right panel*) after 2 h (n = 7–8 mice for each group). **P < 0.01; *P < 0.05, vs. Control (Dunn’s test for 0, 15, and 30 min and Dunnett test for 60, 90, 120 min, and AUC). Results are presented as means ± standard error.

We first examined the effects of long-chain fatty acids (LCFAs)-containing dietary lipids on GLP-1 secretion and glucose homeostasis. We investigated whether LCFAs induce GLP-1 secretion using the mouse intestinal secretin tumor-cell line, STC-1 cells. We observed that linoleic acid (LA), oleic acid (OA), DHA, and EPA significantly induced GLP-1 secretion. Additionally, α-linolenic acid (α-LNA), *cis*-9, *trans*-11 conjugated linoleic acid (CLA1), as well as *trans*-10, *cis*-12 conjugated linoleic acid (CLA3) tended to induce GLP-1 secretion. However, *trans*-9, *trans*-11 conjugated linoleic acid (CLA2), stearic acid (SA), and palmitic acid (PA) had no effects on GLP-1 secretion (Fig. 1a). The incretin GLP-1, a gut hormone that stimulates glucose-induced insulin secretion and inhibits food intake, is secreted from enteroendocrine L cells, which are primarily found in the ileum and colon^20^. We previously reported that the oral administration of the gut microbial PUFA-metabolite, 10-hydroxy-*cis*-12 octadecenoic acid (HYA), strongly induced GLP-1 secretion and improved glucose homeostasis after 1–2 h^21^. We found that plasma GLP-1 levels were significantly induced 2 h after LCFAs (mainly α-LNA, DHA, and EPA) administration (Fig. 1b,c). Hence, 2 h after GLP-1 levels had elevated, we performed an intraperitoneal glucose tolerance test (GTT) and found that administration of DHA and EPA significantly suppressed the increase in blood glucose levels compared to that observed in the control mice (Fig. 1d). Our findings indicated that acute administration of LCFAs, mainly DHA and EPA, promoted GLP-1 secretion and improved glucose homeostasis.

### Polyunsaturated fatty acid-containing dietary lipids improve host metabolism

**Figure 2:**
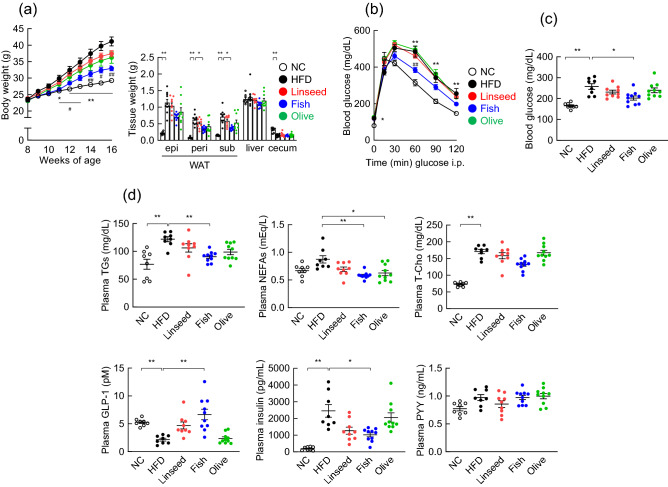
Polyunsaturated fatty acid-containing dietary lipids prevent high fat diet-induced obesity. (**a–d**) C57BL/6J male mice were fed either normal chow (NC), a high fat diet (HFD), or a modified HFD diet [Soybean oil in HFD was replaced with either linseed oil (Linseed), fish oil (Fish), or olive oil (Olive)] for 8 weeks. (**a**) Changes in body and tissue weights. *epi* epididymal, *peri* perirenal, *sub* subcutaneous, *WAT* white adipose tissue. **P < 0.01; *P < 0.05, vs, NC. ^##^*P* < 0.01; ^#^*P* < 0.05, vs, HFD (Dunn’s test for 8, 15, and 16 weeks of age, and tissue weight and Tukey–Kramer test for 9–14 weeks of age). (**b**) ipGTT was evaluated at 15 weeks of age. **P < 0.01; *P < 0.05, vs, NC. ^##^*P* < 0.01, vs, HFD (Dunn’s test for 0, 30, 90, and 120 min and Tukey–Kramer test for 15 and 60 min). (**c,d**) After 5 h fasting, blood glucose, plasma triglycerides, non-esterified fatty acids, total cholesterol, GLP-1, insulin, and PYY levels were measured at the end of the experimental period (n = 7–10 mice for each group). **P < 0.01; *P < 0.05 (Dunn’s test for blood glucose, plasma triglycerides, NEFAs, total cholesterol, GLP-1, and insulin and Tukey–Kramer test for PYY). Results are presented as means ± standard error.

We examined the effects of PUFA-containing dietary lipids on host energy regulation in a mouse model of HFD-induced obesity. We used 8-week-old mice and fed them different PUFA-containing diets as follows; NC (normal chow), HFD (soybean oil), Linseed (Soybean oil in HFD was replaced with linseed oil), Fish (Soybean oil in HFD was replaced with fish oil), and Olive (Soybean oil in HFD was replaced with olive oil), for 8 weeks (Supplementary Table 1). We observed significantly lower body weight of Fish-fed mice compared with HFD-fed mice during development (Fig. 2a). In addition, the white adipose tissue (WAT) fat mass was significantly lower in Fish-fed mice than in HFD-fed mice at 16 weeks of age (Fig. 2a). However, the food intake was similar between HFD-fed and Fish-fed mice (data not shown). Moreover, the HFD-induced impaired glucose tolerance, as determined by GTT, was significantly attenuated in Fish-fed mice compared with HFD-fed mice (Fig. 2b). Furthermore, the blood glucose level of Fish-fed mice was significantly lower than those of HFD-fed mice (Fig. 2c). Additionally, plasma triglycerides (TGs), and non-esterified fatty acids (NEFAs) levels were significantly lower than those of HFD-fed mice, whereas plasma total cholesterol (T-Cho) levels were similar between control and Fish-fed mice (Fig. 2d, *upper panel*). Moreover, we observed significantly higher plasma GLP-1 and significantly lower plasma insulin levels in Fish-fed mice compared to those of HFD-fed mice, whereas plasma PYY levels were similar between control and Fish-fed mice (Fig. 2d, *lower panel*). However, these effects were not observed in Linseed- and Olive-fed mice, suggesting that fish oil replacement improved glucose metabolism, thereby inducing greater resistance to HFD-induced obesity in the presence of DHA and EPA in dietary lipids, but not α-LNA or OA.

### Fish oil replacement regulates systemic inflammation

**Figure 3:**
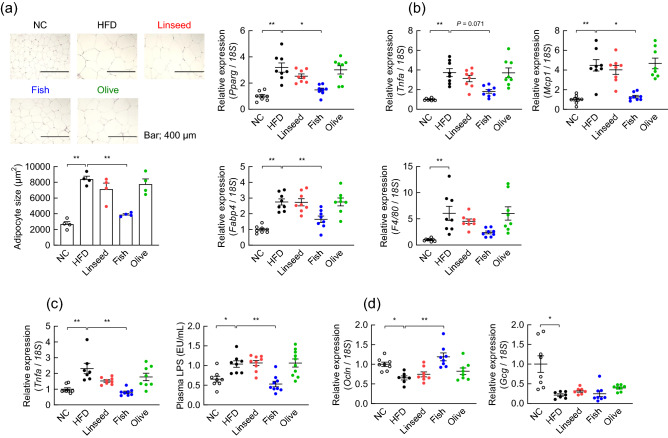
Fish oil suppressed WAT inflammation by enhancing intestinal barrier function. (**a–d**) C57BL/6J male mice were fed either normal chow (NC), a high fat diet (HFD), or a modified HFD diet (Soybean oil in HFD was replaced with either linseed oil (Linseed), fish oil (Fish), or olive oil (Olive)) for 8 weeks. (**a**) Hematoxylin–eosin (H&E)–stained epididymal WAT and the mean area of adipocytes (n = 4 mice for each group). Scale bar; 400 μm. The mRNA expression of *Pparg* and *Fabp4* in the epididymal WAT (n = 8 mice for each group). **P < 0.01; *P < 0.05 (Tukey–Kramer test for adipocytes size and *Fabp4*, and Dunn’s test for *Pparg*). (**b**) The mRNA expression of *Tnfα*, *F4/80*, and *Mcp-1* in the epididymal WAT (n = 8 mice for each group). **P < 0.01; *P < 0.05 (Dunn’s test). (**c**) The mRNA expression of *Tnfα* in the colon (*left panel*) and plasma LPS levels (*right panel*) were measured at the end of the experimental period (n = 7–10 mice for each group). **P < 0.01; *P < 0.05 (Tukey–Kramer test for plasma LPS and Dunn’s test for *Tnfα*). (**d**) The mRNA expression of *Ocln*, and *Gcg* in the colon (n = 7–8 mice for each group). **P < 0.01; *P < 0.05 (Tukey–Kramer test for *Ocln* and Dunn’s test for *Gcg*). Results are presented as means ± standard error.

Obesity, which is a feature of metabolic syndrome, was associated with chronic inflammation in obese subjects and mice^22^. Next, we examined whether fish oil replacement affected adipose and colonic inflammation. We found a significant decrease in WAT adipocyte size in Fish-fed mice (Fig. 3a, *left*). Adipocyte differentiation and increased adipose size are correlated in obesity^23,24^. Next, the mRNA expressions of *Pparg* and *Fabp4* were also decreased in Fish-fed mice (Fig. 3a, *right*). Moreover, we observed significantly decreased mRNA expressions of the inflammatory markers tumor necrosis factor α (*Tnfα*), *F4/80*, and monocyte chemoattractant protein 1 (*Mcp1*) in Fish-fed mice as compared with HFD-fed mice (Fig. 3b). Additionally, the mRNA expression of colonic inflammation marker (*Tnfα*) was also suppressed in Fish-fed mice compared to HFD-fed mice (Fig. 3c, *left*). Ingestion of an HFD increases plasma LPS levels from gram-negative bacteria in the gut, and several studies have shown that HFD-induced obesity is associated with a breach in the intestinal barrier, leading to increases in LPS levels^25,26^. Thus, we investigated the effects of Fish-fed mice on intestinal permeability and barrier function. Fish-fed mice showed significant improvements in intestinal epithelial barrier permeability, as measured by plasma LPS levels (Fig. 3c, *right*). Additionally, the expression of *Ocln*, encoding occludin that plays a key role in maintain the barrier function of tight junction, in the colon was comparable between NC-fed and Fish-fed mice, suggesting a possible role of occludin in influencing gut permeability (Fig. 3d). Moreover, the mRNA expression of *Gcg* (encoding an enteroendocrine cell marker) in the colon was decreased in HFD-fed, Linseed-fed, Fish-fed, and Olive-fed mice compared to the NC-fed mice (Fig. 3d). Altogether, these findings suggested mitigation of increased intestinal permeability, systemic inflammation, and increased GLP-1 secretion in the gut of Fish-fed mice via enhanced intestinal barrier function.

### Fish oil alters the gut microbial composition

**Figure 4:**
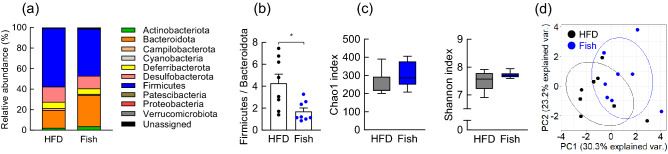
Fish oil altered the gut microbiota composition. (**a**–**d**) C57BL/6J male mice were fed either a high fat diet (HFD) or a modified HFD diet (Soybean oil in HFD was replaced with fish oil (Fish)) for 8 weeks. Gut microbial composition was evaluated for the determination of the relative abundance of microbial phyla (**a**), *Firmicutes* to *Bacteroidota* ratio (**b**), Chao1 and Shannon index at the OTU level (**c**), and principal coordinate analysis (PCoA) at the phylum level (**d**) of mice fed HFD or a modified HFD (Fish) in feces (n = 7–8 mice for each group). *P < 0.05 (Mann–Whitney test).

To assess how dietary lipid sources affect the gut microbial composition, we fed mice isocaloric diets that differed only in lipid composition (either soybean or fish oil) (Supplementary Table 1) for 8 weeks. We analyzed the gut microbial composition using the 16S rRNA gene in the feces of these mice and observed dramatic changes in the microbial ecology based on the dietary lipid type (Fig. 4a–d). The 16S rRNA gene sequencing confirmed that Fish-fed mice had altered the relative abundance of the major phyla constituting the gut microbiota (Fig. 4a). Specifically, in agreement with a previous study^27^, we observed increased Firmicutes and decreased Bacteroidota levels in HFD-fed mice; however, Fish-fed mice had decreased Firmicutes and increased Bacteroidota levels (Fig. 4b). The Chao1 and Shannon indexes were used to evaluate the microbiota richness and diversity. Fish-fed mice had slightly increased alpha diversity compared with HFD-fed mice (Fig. 4c). Additionally, we confirmed that Fish-fed mice had altered gut microbiota composition as compared with HFD-fed mice, indicated by principal coordinate analysis (PCoA) based on taxonomic datasets (Fig. 4d).

### The metabolic effects of fish oil were abolished in antibiotics-treated mice

**Figure 5:**
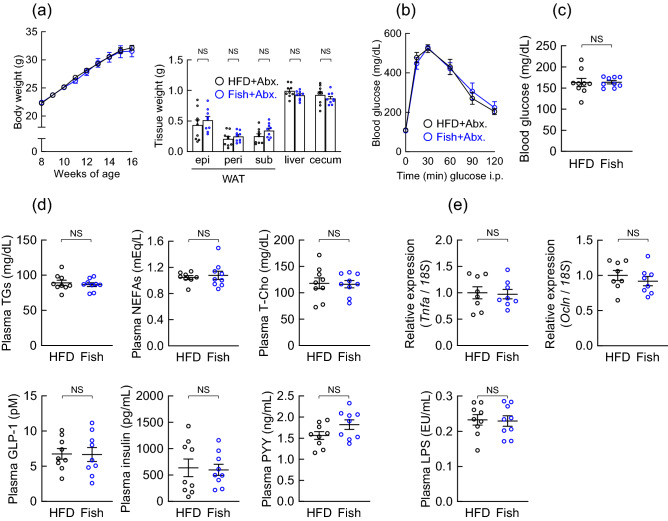
Fish oil improved metabolic conditions were attenuated in antibiotic-treated mice. (**a–e**) C57BL/6J male mice were fed either a high fat diet (HFD) or a modified HFD diet (Soybean oil in HFD was replaced with fish oil (Fish)) and treated with antibiotics (Abx.) for 8 weeks. (**a**) Changes in body and tissue weights. *epi* epididymal, *peri* perirenal, *sub* subcutaneous, *WAT* white adipose tissue. (**b**) ipGTT was evaluated at 15 weeks of age. (**c,d**) After 5 h fasting, blood glucose, plasma triglycerides, non-esterified fatty acids, total cholesterol, GLP-1, insulin, and PYY levels were measured at the end of the experimental period. (**e**) The mRNA expression of *Tnfα* and *Ocln* in the colon (*upper panel*) and plasma LPS levels (*lower panel*) were measured at the end of the experimental period (n = 7–9 mice for each group). Results are presented as means ± standard error. *NS* not significant.

Finally, to determine whether the metabolic effects of the Fish oil-containing diet depend on the gut microbiota, we further assessed the effects of the Fish oil-containing diet in antibiotics-treated mice (Abx.). Consistent with the findings of a previous study^28,29^, we observed an increase in cecal tissue weight and a decrease in the total bacterial levels in HFD- and Fish-fed, Abx.-treated mice (Supplementary Fig. 1). Similar to Figs. 2 and 3, HFD induced an increase in body and tissue weight, glucose intolerance, and colonic inflammation, while Fish-fed mice restored these metabolic parameters and inflammation (data not shown). However, body and tissue weight, glucose tolerance, and blood glucose levels were comparable between HFD- and Fish-fed in Abx.-treated mice (Fig. 5a–c). Additionally, levels of the plasma metabolic parameters (TGs, NEFAs, T-Cho, GLP-1, and insulin) were similar between HFD- and Fish-fed in Abx.-treated mice (Fig. 5d). Moreover, by modulating colonic inflammation and permeability, mRNA expressions of *Tnfα* and *occludin*, and plasma LPS levels were comparable between HFD- and Fish-fed in Abx.-treated mice (Fig. 5e). In white adipose tissues, adipocyte size and mRNA expressions of *Pparg*, *Fabp4*, *Tnfα*, *F4/80*, and *Mcp1* were comparable between HFD-fed and Fish-fed in Abx.-treated mice (Supplementary Fig. 2). Thus, these results suggested that the metabolic benefits of fish oil depend partially upon the gut microbiota.

## Discussion

Polyunsaturated fatty acids, especially DHA and EPA, improve glucose tolerance and obesity. However, the mechanisms underlying the beneficial effects of dietary PUFA-enriched oil but not FFAs on obesity remain unclear. In this study, we report that PUFA containing a high level of fish oil was efficacious against body and tissue weights by improving glucose metabolism and modulating gut microbiota. These metabolic benefits of fish oil-enriched PUFA were associated with the gut microbial composition and their effects on promoting intestinal barrier function (Supplementary Fig. 3).

PUFAs as FFAs exert several beneficial effects, improving tight junction permeability, facilitating epithelial cell proliferation, changing gut microbial composition, and gut hormone secretion in the colon^21,30^. Additionally, numerous studies demonstrated that PUFAs, mainly DHA and EPA, promote the secretion of GLP-1^12,14,31^. Our data suggested that DHA and EPA-stimulated STC-1 cells and Fish-fed mice specifically promoted GLP-1 secretion. Although α-linolenic acid and oleic acid also tended to promote GLP-1 secretion in STC-1 cells, Linseed (rich in α-linolenic acid) and Olive (rich in oleic acid)-fed mice did not exhibit increased GLP-1 secretion. FFAs in dietary lipids are produced from lipid precursors by the action of lipases. Increased circulating LPS suppresses lipase activity, favoring the accumulation of triglycerides in the intestine and other organs^32^. Moreover, the lipid type entering the small intestine and its absorption through the stomach wall directly affects the amount and type of lipid entering the colon. Indeed, unsaturated FAs are more easily absorbed than saturated FAs. The mechanisms by which dietary fatty acids affect the gut microbiota are not clear. Although most consumed fatty acids are absorbed in the small intestine, a small amount passes through the gastrointestinal tract and may therefore directly modulate colonic microbiota composition. Moreover, lipid digestion in the stomach and absorption into the small intestine affects the amount and type of lipids entering the colon. The mechanistic details of how dietary fatty acids affect the gut microbiota are not known. The basal levels of the incretin hormone, GLP-1 are elevated in mice lacking gut microbiota, suggesting that in the absence of gut microbiota, increased colonic-derived GLP-1 slows intestinal transit, allowing more time for nutrient absorption^33^. In contrast, gut microbial metabolites (e.g., SCFAs, secondary bile acids, and HYA) promote GLP-1 secretion^21,34,35^. These findings exemplify how microbial contribution to systemic energy supply affects host gene expression and physiology in the gut. Therefore, it is necessary to examine the location where the LCFAs in ingested dietary lipids are converted to FFAs thereby, affecting physiological functions.

Chronic HFD resulting in obesity leads to a leaky intestinal epithelial barrier^25,26^. Consequently, LPS can enter the systemic circulation and induce chronic inflammation that leads to glucose intolerance^36^. We showed that fish oil feeding reduced circulating LPS levels and systemic inflammation, suggesting that fish oil might be conducive to maintaining intestinal epithelial barrier function and gut microbial homeostasis, thus improving glucose tolerance. Moreover, the 16S rRNA gene sequencing analysis confirmed that the gut microbial composition in Fish-fed mice was altered compared to that in the HFD-fed mice. Supplemental Table 1 shows that saturated fatty acids that are enriched in lard exhibit toxic influences the inhibiting bacterial growth, antibacterial activity such as lysis and solubilization of bacterial cell membranes, and inhibition of ATP production^37–39^. On the other hand, a comparison of mice on a variety of diets (low-fat diet and diets containing high levels of saturated fatty acids, ω6 PUFA, or ω3 PUFA) showed that diets with saturated fatty acids or ω6 PUFAs induced weight gain, however, increased insulin resistance, colonic permeability, and mesenteric fat inflammation were only induced saturated fats^8^. The gut microbiota composition of mice fed a low-fat diet and a ω3 PUFA diet differed from the other groups, whereas the gut microbiota of mice fed a saturated fat diet and a ω6 PUFA diet were similar. Therefore, gut microbial functions and FFA composition in dietary lipids associated between diet, gut microbiota structure, and dyslipidemia should be studied in large human cohorts to develop therapeutic strategies for treatment of metabolic disorders.

In conclusion, DHA and EPA-rich fish oil could ameliorate HFD-induced obesity and glucose intolerance partly by regulating the gut microbiome, modulating gut permeability, and promoting GLP-1 secretion. DHA- and EPA-stimulated GLP-1 secretion in enteroendocrine cells might play an important role in linking diet, gut microbiome, and host metabolism. This study provides a new mechanism underlying the function of DHA and EPA in decreasing blood glucose levels in obese individuals.

## Materials and methods

### Cell culture

STC-1 cells (murine enteroendocrine cells) were purchased from American Type Culture Collection (ATCC; Manassas, VA, USA) and cultured in Dulbecco’s modified Eagle medium (DMEM) containing 5% fetal bovine serum (FBS) and 15% horse serum and were maintained at 37 °C with 5% CO_2_. For measurement of GLP-1 secretion, cells were plated in 24-well plates (3 × 10^5^ cells/well) and cultured for 48 h before inducing GLP-1 secretion. After 48 h, the culture media was collected, and serum-free media was added followed by incubation for 90 min. Each well was treated with FAs [200 μM; linolenic acid (LA), oleic acid (OA), α-linolenic acid (α-LNA), docosahexaenoic acid (DHA), eicosapentaenoic acid (EPA), *cis*-9, *trans*-11-conjugated linoleic acid (CLA1), *trans*-9, *trans*-11-conjugated linoleic acid (CLA2), *trans*-10, *cis*-12-conjugated linoleic acid (CLA3), stearic acid (SA), or palmitic acid (PA)] for 1 h, and the supernatant was collected in the presence of a dipeptidyl peptidase IV (DPP-IV) inhibitor.

### Animal study

C57BL/6J male mice were purchased from Japan SLC (Shizuoka, Japan) and housed under a 12-h light–dark cycle. All experimental procedures involving mice were performed according to protocols approved by the Committee on the Ethics of Animal Experiments of the Tokyo University of Agriculture and Technology (permit No. R4-147) and the Animal Welfare Act Regulations and Guide for the Care and Use of Laboratory Animals. The study has been reported in accordance with the ARRIVE guidelines^40^.

The glucose tolerance test (GTT) and GLP-1 secretion measurement were performed after acute administration of FAs (LA, OA, α-LNA, DHA, or EPA). For GTT, 7-week-old C57BL/6J male mice were administered FAs (1 g/kg of body weight) dissolved in 0.5% CMC (Carboxymethyl cellulose ammonium, Nacalai Tesque, Kyoto, Japan) by gavage following a 16-h fast period^21^. After 2 h, mice were administered glucose (2 g/kg of body weight) intraperitoneally. Plasma glucose concentration in the tail vein was monitored before glucose injection and at 15-, 30-, 60-, 90-, and 120-min post-injection. For GLP-1 measurement, 8-week-old C57BL/6J male mice were administered FFAs (1 g/kg of body weight) dissolved in 0.5% CMC by gavage following a 16-h fast period, and plasma samples were collected from the vena cava after 2 h.

For long-term treatment, 7-week-old C57BL/6 J mice were acclimatized for 1 week with a normal chow (NC, CE-2; CLEA, Tokyo, Japan). After acclimatization, mice were randomly allocated into each treatment group, and fed either NC, an HFD (D12492: Research Diets, New Brunswick, NJ, USA), or a modified HFD diet [Soybean oil in HFD was replaced with either linseed oil (Sigma-Aldrich), fish oil (Sigma-Aldrich), or olive oil (Sigma-Aldrich)] for 8 weeks. The compositions of the diets are provided in Supplementary Table 1. Antibiotic-treated mice were given a cocktail of antibiotics [0.5 g/L ampicillin (Wako Pure Chemical Co. Ltd., Osaka, Japan), 0.2 g/L vancomycin (Wako), 0.5 g/L neomycin (Wako) and 0.5 g/L metronidazole (Wako)] in their drinking water. Diets and water were provided ad libitum throughout the experiment^41^. After 7 weeks of treatment, mice were made to fast for 16 h followed by the GTT.

### Biochemical analyses

Blood glucose concentrations were measured using a One Touch Ultra device (LifeScan, Milpitas, CA, USA). The concentrations of plasma triglycerides (LabAssay Triglyceride; Wako), total cholesterol (LabAssay Cholesterol; Wako), non-esterified fatty acid (LabAssay NEFA; Wako), Peptide YY (Mouse/Rat PYY ELISA Kit, Wako Pure Chemical Co. Ltd., Osaka, Japan), GLP-1 (active) ELISA kit (Merck Millipore, Billerica, MA, USA), and insulin ELISA Kit (Shibayagi, Gunma, Japan) were measured according to manufacturer instructions. For GLP-1 measurement, plasma samples and culture media were treated with a DPP-IV inhibitor (Merck Millipore, Billerica, MA, USA) to prevent degradation of active GLP-1. Bacterial endotoxin levels were evaluated by a limulus amebocyte lysate chromogenic endpoint assay, according to the manufacturer’s instructions (Hycult Biotech, Wayne, PA, USA).

### Histology

Epididymal white adipose tissues were fixed in 10% neutral-buffered formalin, embedded in paraffin, and sectioned (7 μm). Hematoxylin and eosin staining was performed using standard techniques^42^, with minor modifications. To measure adipocyte area, the diameters of ≥ 20 cells from 4 sections in each group were measured using ImageJ software (National Institutes of Health, Bethesda, MD, USA). The results were expressed as the average of > 10 fields examined.

### RNA extraction and qRT-PCR

Total RNA was isolated from epididymal WAT and colon using a RNAiso Plus reagent (TaKaRa, Shiga, Japan). cDNA was transcribed using RNA as templates and Moloney murine leukemia virus reverse transcriptase (Invitrogen, Carlsbad, Cam USA). Quantitative reverse transcriptase (qRT)-PCR analysis was performed using the StepOnePlus real-time PCR system (Applied Biosystems, Foster City, CA, USA) with TB Green Premix Ex Taq II (TaKaRa). The reaction was performed at 95 °C for 30 s, followed by 40 cycles of 95 °C for 5 s, 58 °C for 30 s and 72 °C for 1 min. The dissociation stage was analyzed at 95 °C for 15 s, followed by 1 cycle of 60 °C for 1 min and 95 °C for 15 s. Primer sequences were as follows: *Pparg*, 5′-TCAGCTCTGTGGACCTCTCC-3′ (forward) and 5′-ACCCTTGCATCCTTCACAAG-3′ (reverse); *Fabp4*, 5′-GATGCCTTTGTGGGAACCTGG-3′ (forward) and 5′-CTGTCGTCTGCGGTGATTTC-3’(reverse); *Tnfa*, 5′-TCGTAGCAAACCACCAAGTG-3′ (forward) and 5′-CTTTGAGATCCATGCCGTTG-3′ (reverse); *F4/80*, 5′-GGAGGATGGGAGATGGACAC-3′ (forward) and 5′-ACAGCACGACACAGCAGGAA-3′ (reverse); *Ocln*, 5′-CACACTTGCTTGGGACAGAG-3′ (forward) and 5′-TAGCCATAGCCTCCATAGCC-3′ (reverse); *Mcp1*, 5′-AATCTGAAGCTAATGCATCC-3′ (forward) and 5′-GTGTTGAATCTGGATTCACA-3′ (reverse); *Gcg*, 5′-TGGACTCCCGCCGTGCCCAA-3′ (forward) and 5′-CGACTTCTTCTGGGAAGTCTCGCCT-3′ (reverse); and *18S*, 5′-ACGCTGAGCCAGTCAGTGTA-3′ (forward) and 5′-CTTAGAGGGACAAGTGGCG-3′ (reverse).

### Gut microbial composition

Fecal DNA was extracted from frozen samples using the FastDNA SPIN kit for feces (MP Biomedicals, Santa Ana, CA, USA) according to manufacturer instructions^20^. Partial 16 S rRNA gene sequences were amplified targeting the hypervariable regions v4 using primers 515 F (5′-TCGTCGGCAGCGTCAGATGTGTATAAGAGACAGGTGYCAGCMGCCGCGGTAA-3′) and 806 R (5′-GTCTCGTGGGCTCGGAGATGTGTATAAGAGACAGGGACTACHVGGGTWTCTAAT-3′). Amplicons generated from each sample were subsequently purified using AMPure XP (Beckman Coulter, Brea, CA, USA). The 16S rRNA sequence data generated by the MiSeq sequencer (Illumina, San Diego, CA, USA) using the MiSeq Reagent kit (version 3.0; 600 cycles) were processed by the quantitative insights into microbial ecology (QIIME 2. 2022.2.0; http://qiime.org/) pipeline. Data analysis was performed using MiSeq Reporter software with the SILVA database (Illumina). Diversity analyses were performed using the QIIME script core_diversity_analyses.py. The statistical significance of sample groupings was assessed using permutational multivariate analysis of variance (QIIME script compare_categories.py). The 16S rRNA sequencing data have been deposited into the DDBJ under the accession No. DRA015131.

Quantitative PCR analysis was performed with using SYBR Premix Ex Taq II (TAKARA) and StepOneTM real time PCR system (Applied Biosystems) as previously described^41^. Bacterial primer sequences are as follows; Universal, 5′-CRAACAGGATTAGAACCCT-3′ (forward) and 5′-GGTAAGGTTCCTCGCGTAT-3′ (reverse).

### Statistical analysis

All values are presented as the mean ± standard error. Data were analyzed using commercially available statistical software: Prism version 9.4.1 (GraphPad Software, La Jolla, CA, www.graphpad.com). Data normality was checked by using the Shapiro–Wilk test. For normal data, equal-variance test was carried out by F test (for comparing two groups) or Bartlett’s test (for comparing more than 3 groups). When comparing 2 groups, either 2-tailed unpaired *t*-test (for parametric data) or Mann–Whitney test (for nonparametric data) was used. When comparing more than 3 groups, one-way analysis of variance (ANOVA) (for parametric data) or Kruskal–Wallis test (for nonparametric data) was carried out, followed by post-hoc multiple comparisons tests either by Dunnett, Tukey–Kramer, or Dunn’s test. *P*-values from these multiple comparison tests were corrected and reported as adjusted *P* values. Statistical significance was set at *P* < 0.05.

## Supplementary Information

Supplementary Information.

## Data Availability

The datasets used and/or analyzed during the current study are available from the corresponding author on reasonable request. The 16S rRNA sequencing data have been deposited into the DDBJ under the Accession No. DRA015131 (https://ddbj.nig.ac.jp/resource/sra-submission/DRA015131).
