# Supplementary material for: Polyunsaturated fatty acids-rich dietary lipid prevents high fat diet-induced obesity in mice
Source: Sci Rep. 2023 Apr 5;13:5556. doi: 10.1038/s41598-023-32851-7 (PMC10076282; doi:10.1038/s41598-023-32851-7)
Supplement: Supplementary file 1 — Supplementary Information. [file 41598_2023_32851_MOESM1_ESM.docx]

**Polyunsaturated fatty acids-rich dietary lipid prevents high fat diet-induced obesity in mice.**

Yuri Haneishi^1#^, Yuma Furuya^1#^, Mayu Hasegawa^1^, Hitoshi Takemae^2^, Yuri Tanioka^3^,

Tetsuya Mizutani^2^, Mauro Rossi^4^, Junki Miyamoto^1*^

^1^Department of Applied Biological Science, Graduate School of Agriculture, Tokyo University of Agriculture and Technology, Fuchu-shi, Tokyo 183-8509, Japan, ^2^Center for Infectious Diseases Epidemiology and Prevention Research: CEPiR, Tokyo University of Agriculture and Technology, Fuchu-shi, Tokyo 183-8509, Japan, ^3^Department of International Food and Agricultural Science, Faculty of International Food and Agricultural Studies, Tokyo University of Agriculture, Setagaya-ku, Tokyo 156-8502, Japan, ^4^ Institute of Food Sciences, CNR, via Roma 64, 83100 Avellino, Italy.

^#^Contributed equally

^*^Corresponding author: J.M. ([m-junki@go.tuat.ac.jp](mailto:m-junki@go.tuat.ac.jp))

**Supplementary Figure 1. Changes in gut microbial composition in antibiotic-treated mice.**

C57BL/6 J male mice were fed either a high fat diet (HFD) or a modified HFD diet (Soybean oil in HFD was replaced with fish oil (Fish)) and treated with antibiotics (Abx.) for 8 weeks. Cecal tissue weight (*left*) and the gut microbiota composition were determined by performing qRT-PCR for all the bacterial populations (*right*) (n = 8–10 mice for each group). Cecal tissues in HFD-fed and Fish-fed mice are referred to in figure 2a.

**Supplementary Figure 2. Fish oil improved inflammation in WAT by gut microbiota.**

(**a**–**b**) C57BL/6 J male mice were fed either a high fat diet (HFD) or a modified HFD diet (Soybean oil in HFD was replaced with fish oil (Fish)) and treated with antibiotics (Abx.) for 8 weeks. (**a**) Histological analysis with hematoxylin–eosin (H&E)–stained epididymal WAT and the mean size of adipocytes (n = 4 mice for each group). Scale bar, 400 μm. The mRNA expression of *Pparg* and *Fabp4* in the epididymal WAT (n = 9 mice for each group). (**b**) The mRNA expression of *Tnfα*, *F4/80*, and *Mcp1* in the epididymal WAT (n = 9 mice for each group). Results are presented as means ± standard error. NS; not significant.

**Supplementary Figure 3. Polyunsaturated fatty acids-rich Fish oil influenced metabolic conditions and ameliorated HFD-induced obesity through modulating gut microbiota.**

The mechanism by which dietary PUFAs-rich Fish oil contributes to host resistance to obesity

**Supplementary Table 1. Diet compositions.**
